# Effects of Colostrum-Period Fecal Microbiota Transplantation on Growth Performance, Immune Function and Gut Microbiota in Pre-Weaned Calves

**DOI:** 10.3390/ani16193088

**Published:** 2026-10-01

**Authors:** Jingkun Zhang, Yaning Sun, Xintong Li, Duo You, Zhanhe Zhang, Ming Xu, Donglin Wu

**Affiliations:** College of Animal Science, Inner Mongolia Agricultural University, Hohhot 010018, China; 17624075086@163.com (J.Z.); 13624722788@163.com (Y.S.); 17548951390@163.com (X.L.); youduo202608@163.com (D.Y.); nddky@emails.imau.edu.cn (Z.Z.)

**Keywords:** average daily gain, diarrhea incidence, FMT administration route, IgG, Interleukin

## Abstract

Neonatal calf diarrhea caused by intestinal microbial dysbiosis severely restricts rearing efficiency, and fecal microbiota transplantation (FMT) emerges as a promising antibiotic-free strategy for regulating gut homeostasis. Nevertheless, to the best of our knowledge, the effects of colostrum-period FMT and the functional differences between colonic and colostrum delivery routes remain poorly characterized in pre-weaned dairy calves. We hypothesized that colostrum-period FMT would accelerate gut microbiota maturation, improve immune status and reduce diarrhea incidence in pre-weaned calves, with delivery-route-dependent effects. This study aimed to investigate how two colostrum-phase FMT delivery routes shape growth performance, serum immune profiles and gut microbiota in neonatal Holstein calves. Sixty newborn calves were randomly assigned to three groups (*n* = 20 per group): control (no FMT), colon-FMT (colonic perfusion), and colostrum-FMT (FMT mixed with colostrum). Donor feces were homogenized with sterile saline at a 1:9 ratio for FMT suspension preparation, and the trial lasted 56 days. For growth performance, the colon-FMT calves exhibited significantly higher average daily gain from D29 to D56 (*p* < 0.05). In terms of intestinal health, the colostrum-FMT group exhibited lower diarrhea incidence during D15-28 (*p* < 0.05) but markedly higher diarrhea rates during D29-56 (*p* < 0.01). For serum immunity, colon-FMT calves had elevated serum IgG at D14 (*p* < 0.05). At D56, the concentrations of IL-2 (*p* < 0.01) and IL-4 (*p* = 0.05) were significantly lower in the colostrum-FMT group than the colon-FMT group; no intergroup differences were detected for other serum cytokines and immunoglobulins. 16S rRNA gene sequencing of D28 fecal samples revealed that the colon-FMT group possessed a significantly higher relative abundance of *Lactobacillus* compared with the other two treatments (*p* < 0.05). Collectively, colonic FMT is associated with remodeling intestinal microbiota by enriching beneficial *Lactobacillus*, improved late-stage growth and sustained long-term intestinal health in pre-weaned calves, whereas colostrum-mediated FMT only provides transient protection against early diarrhea.

## Simple Summary

Young dairy calves frequently develop serious diarrhea triggered by disrupted gut microbiota communities, which hinders growth performance and increases dependence on antibiotics within the dairy industry. Transferring healthy gut microbiota from donor animals offers a promising drug-free solution to stabilize calf intestinal health. This study tested two methods to provide healthy gut microbiota to newborn calves during their first feeding stage: direct administration into the colon and mixing the microbiota with first milk. Researchers monitored calf weight gain, the occurrence of diarrhea, immune conditions and intestinal bacteria changes over a 56-day feeding trial. The colon delivery method improved calf weight gain in the later raising stage, reduced diarrhea risks and boosted the abundance of helpful gut microbiota. By contrast, supplying microbiota mixed with first milk only lowered diarrhea rates in the early phase, yet led to more frequent diarrhea as calves grew older. This trial firstly validates colonic delivery as an antibiotic-free approach for regulating neonatal calf gut microbiota, growth and immunity. These practical outcomes guide dairy producers to select more effective strategies to prevent calf gastrointestinal diseases, support sustainable calf rearing, and help cut unnecessary antibiotic use in modern dairy production systems.

## 1. Introduction

The gut microbiota acts as a critical “metabolic organ” that modulates host metabolism, immunity and intestinal homeostasis across animals and humans [1,2,3]. Symbiotic gut bacteria maintain immune balance; microbial dysbiosis readily triggers intestinal inflammation and metabolic disorders [4]. Given that dysbiosis is a major driver of enteric disease, restoring commensal communities via fecal microbiota transplantation (FMT) represents a logical corrective intervention.

Fecal microbiota transplantation (FMT) restores disrupted gut flora by transferring fecal microbes from healthy donors. Dating back to Ge Hong’s ancient Jin Dynasty medical records, FMT has long been applied to treat severe diarrhea and toxic enteritis. Modern clinical trials further validated its outstanding efficacy against human recurrent Clostridium difficile infection, irritable bowel syndrome and Crohn’s disease [5,6,7,8,9]. While FMT’s growth and intestinal benefits have been documented in humans [7,8,10,11,12,13] and piglets [14,15,16], its regulatory effects on neonatal dairy calves remain unclear to our knowledge.

Neonatal dairy calves exhibit well-orchestrated gut microbiota establishment after birth. The sterile gastrointestinal tract is rapidly colonized by microbes from dams, colostrum and farm surroundings, with communities maturing into a stable ruminant-type microbiome within the first weeks of life [17]. Beyond passive immunity, colostrum delivers commensal bacteria, oligosaccharides and bioactive compounds that modulate gut microbial assembly and mucosal immune maturation [18]. Early-life gut microbiota exerts long-term programming effects linked to diarrhea susceptibility, immune competence, growth and lifelong production in dairy cattle [19]. Due to high postnatal intestinal plasticity, this time window offers a promising opportunity for FMT to shape microbiome maturation toward a health-beneficial state.

Neonatal gastrointestinal disease is a global challenge for calf production. Digestive disorders account for 32% of pre-weaned calf mortality on U.S. farms [20], whereas the corresponding proportion reaches 40% or higher among Chinese herds, with large variation depending on farm management conditions. Calf diarrhea remains a persistent bottleneck restricting rearing efficiency [21,22]. Early gut microbiota succession in newborn calves has been insufficiently characterized, and gut flora has been well-established to shape neonatal immune development [23,24,25,26,27,28,29,30]. Because early microbiota maturation shapes both immune competence and disease risk, interventions targeting this window may alter long-term calf outcomes, motivating empirical testing of FMT in neonatal calves.

This study represents the first trial comparing two FMT delivery routes during the colostrum period in neonatal dairy calves. It aims to explore how colostrum-period FMT modulates growth performance, serum immune indices and intestinal microbiota and to provide insights into potential mechanism for improving calf intestinal health. We hypothesized that colostrum-period FMT would accelerate gut microbiota maturation, enhance host immune indices and reduce diarrhea risk and that biological outcomes would differ depending on whether FMT was delivered via colonic perfusion or mixed within colostrum. Our findings will offer theoretical support for applying FMT as a novel strategy to prevent calf gastrointestinal diseases.

## 2. Materials and Methods

### 2.1. Preparation of Fecal Microbiota Suspension

Donor screening: Healthy adult cattle with no historical illness and who were diarrhea-free for at least 7 days were selected as donors. Fresh rectal feces were screened using the QUICK^®^ DIAR 7 rapid antigen test kit (CEVA Santé animale, Libourne, France), following the manufacturer’s instructions); all pathogens (rotavirus, coronavirus, K99 *E. coli*, *Cryptosporidium*, *Clostridium perfringens*) tested negative. Fecal inoculum was pooled from 5 eligible donor cattle to reduce individual donor microbiota variability.

Suspension preparation: Fresh feces were collected by rectal stimulation and transported under refrigeration immediately. Fecal material was homogenized with sterile normal saline at a 1:9 ratio, fully mixed and left to settle for 5–8 min. The mixture was double-filtered through four layers of sterile gauze, aliquoted into 50 mL centrifuge tubes and stored at −20 °C. Before transplantation, frozen suspensions were thawed in a 37 °C water bath to retain microbial viability.

### 2.2. Experimental Design and Animal Management

A single-factor completely randomized design was adopted to investigate the effects of colostrum-period FMT on growth performance, serum indices and gut microbiota of pre-weaned Holstein calves. Sample size (*n* = 20 per group) was selected based on empirical medium-to-large treatment effects reported in previous calf FMT studies focusing on diarrhea and growth endpoints [31,32]. Those studies used smaller group sizes (10–14 calves per group), whereas we increased the number of calves to accommodate potential animal dropout and higher biological variability under commercial farm conditions.

Sixty healthy newborn Chinese Holstein calves (initial BW: 40.26 ± 5.77 kg) were equally and randomly allocated to three groups (*n* = 20, balanced sex ratio): control (no fecal suspension), colon-FMT (colonic perfusion), colostrum-FMT (perfusion via colostrum). Calves in the FMT groups received two 50 mL fecal suspension perfusions: the first at the first colostrum feeding, and the second 24 h thereafter. The volume and two-dose schedule were adapted from published calf FMT protocols [31,33]; two repeated administrations were intended to improve microbial engraftment probability in neonatal recipients.

For the colonic-FMT perfusion procedure, a soft-tipped, lubricated rectal catheter was inserted approximately 15–20 cm into the distal colon. The calves received no sedation; they were only gently restrained manually, with no additional restrictive physical restraint. A total of 50 mL thawed FMT suspension was slowly infused, followed by a small air flush to clear catheter dead-volume. The calves were held in a raised-hindquarter position for 2–3 min post-infusion to minimize immediate reflux. The trial lasted 56 days. All calves were fed pasteurized colostrum (10% birth weight) within 1 h post-birth, followed by a second colostrum feeding at 4 h. Pasteurized milk was provided twice daily at 08:00 and 17:00 thereafter.

Personnel assessing the fecal score and measuring body weight were blinded to group assignment to reduce assessment bias. Sampling time points included D1, D7, D14, D28 and D56. Four sequential sampling time points (D1, D7, D14 and D28) were prioritized for the early pre-weaning phase, representing a critical developmental window for calf gut microbiota; D56 was retained as the late pre-weaning endpoint to assess the longer-term outcomes of FMT. No intermediate sampling was conducted between D28 and D56 due to concerns over additional calf stress, heavy workload and experimental costs.

### 2.3. Sample Collection and Indicator Determination

Calf body weight (BW) was measured pre-feeding at D1, D7, D14, D28 and D56, and the stage-specific average daily gain (ADG) was calculated. Fecal scores (1–4 scale; reference: Larson et al. [34]) were recorded daily to assess calf intestinal health. Calves with a fecal score ≥ 3 were recorded as diarrheic.

Blood samples were harvested via jugular venipuncture pre-feeding at D1, D7, D14, D28 and D56. Specimens were collected into serum coagulation tubes, centrifuged at 1800× *g* for 15 min at 4 °C to separate serum and stored at −80 °C. Concentrations of serum IgG, IgA, IgM, IL-2, IL-4, IL-6 and IL-10 were determined using commercial ELISA kits (Nanjing AngleGene BioTechnology Co., Ltd., Nanjing, China).

Fresh feces were collected via rectal stimulation on D28, transferred to sterile cryovials, snap-frozen in liquid nitrogen and stored at −80 °C. All fecal samples were delivered to Shanghai Majorbio Bio-pharm Technology Co., Ltd. (Shanghai, China) for 16S rRNA gene sequencing to analyze gut bacterial diversity and taxon abundance. Microbiota profiling was only performed for D28 fecal samples; no sequencing was conducted at D56.

### 2.4. Statistical Analysis

SPSS 26.0 and R 4.2.1 were used for data analysis. For repeatedly measured BW, serum immunological and cytokine data, linear mixed-effect models were fitted with treatment, time and treatment-by-time interaction as fixed factors, and individual calf as the random intercept. One-way ANOVA with the Tukey-HSD multiple-comparison test was applied for single-time-point comparisons. *p* ≤ 0.05 indicated a significant difference, *p* = 0.05 was interpreted as borderline significance, and *p* ≤ 0.01 indicated a highly significant difference.

ASV-derived alpha-diversity metrics were compared by one-way ANOVA. Beta diversity was visualized by PCoA based on the Bray–Curtis distance, and PERMANOVA (999 permutations) tested community-level differences. LEfSe-LDA (threshold LDA > 2) was applied to identify signature bacterial genera. Correlation analyses between bacterial genera and growth phenotypes (ADG, BW) were performed for exploratory purposes, without multiple-testing correction.

## 3. Results

### 3.1. Growth Performance

No significant differences were detected in calf BW at all sampling days or ADG across D1–D7, D8–D14, D15–D28 and the whole trial (D1–D56, Table 1) among the three groups (*p* > 0.05). Nevertheless, ADG during D29–D56 differed markedly (*p* = 0.01), with the colon group showing significantly higher ADG (1.23 kg) than the control (1.19 kg) and colostrum (0.98 kg) groups. These data indicate that colonic FMT was associated with improved late-stage growth in pre-weaned calves, wherea*s* colostrum-delivered FMT exerted no such beneficial effects.

### 3.2. Intestinal Health

No significant intergroup differences were found in fecal consistency scores and diarrhea rate during D1-7 and D8-14 (*p* > 0.05, Table 2). Over D15-28, the colostrum-FMT group exhibited a lower diarrhea rate (0.08) than the other two groups (control, 0.22; colon, 0.20; *p* = 0.01). Conversely, during D29-56, colostrum-FMT calves had higher fecal scores (1.77) and diarrhea incidence (0.17, *p* < 0.01), while the colon-FMT group maintained favorable intestinal health status equivalent to the control.

### 3.3. Blood Immune Indices

No intergroup differences were observed for serum IgA and IgM across all time points (*p* > 0.05, Table 3). Serum IgG only differed at D14, where the colon-FMT group tended to have higher IgG levels than the control and colostrum-FMT groups (*p* = 0.05).

No significant intergroup differences in IL-6 and IL-10 were detected at all time points (*p* > 0.05, Table 4). Concentrations of IL-2 and IL-4 did not differ among groups before D56 (*p* > 0.05), while the colostrum-FMT group had lower levels of IL-2 (*p* < 0.01) and IL-4 (*p* = 0.05) compared with the control and colon-FMT groups at D56; the difference in IL-4 represented a statistical tendency.

### 3.4. Intestinal Flora

We analyzed fecal bacterial communities to evaluate the effects of different FMT administration modes on the gut microbiota of pre-weaned calves. A total of 3,849,427 clean reads were obtained. Good’s coverage indicated that the sequencing depth was sufficient for microbial-diversity analysis, with no significant differences in sequencing depth among the three groups.

**Figure 1:**
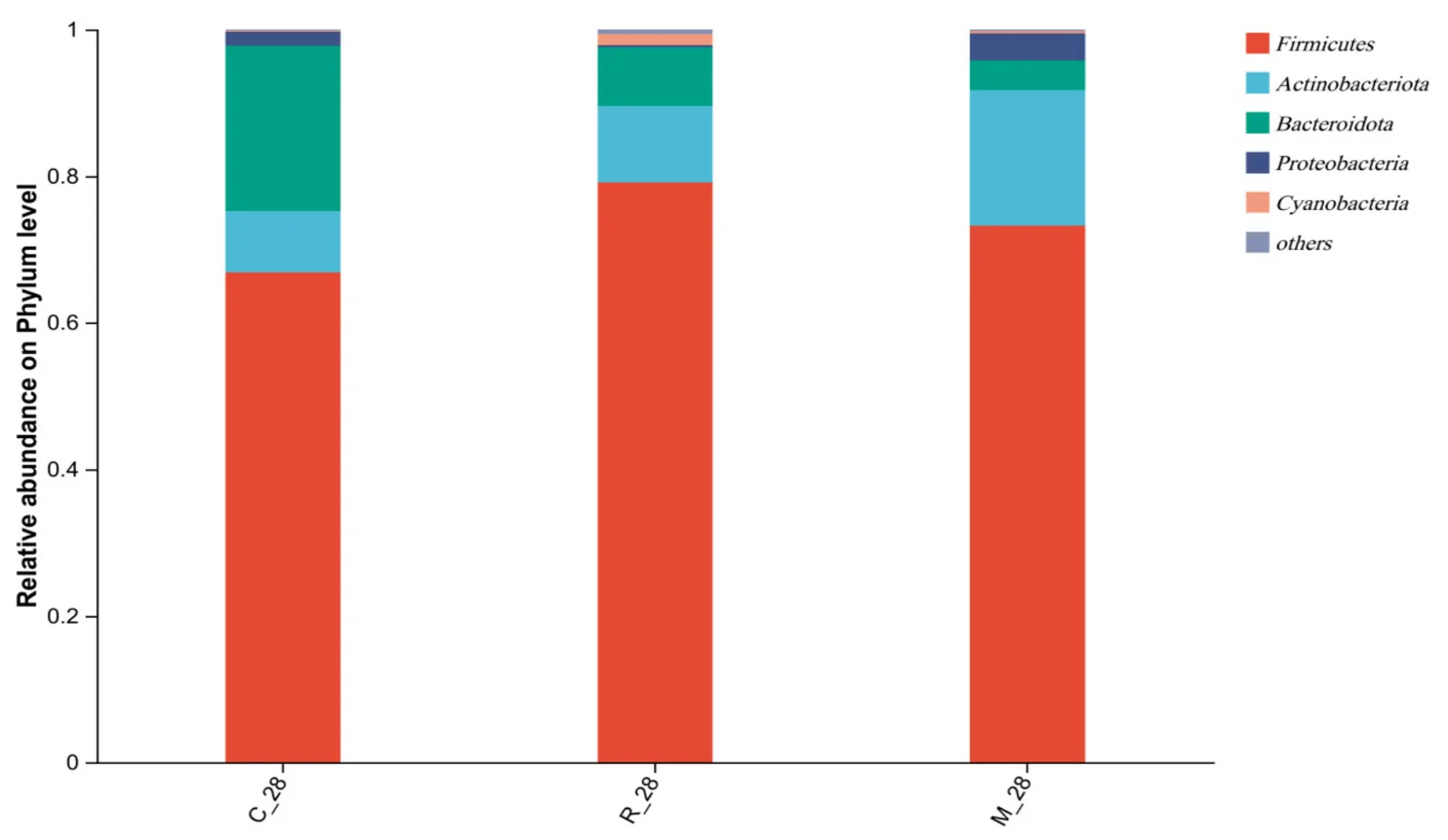
Relative abundance of microbiota at the phylum level among groups. C: control; M: colon-FMT; R: colostrum-FMT.

**Figure 2:**
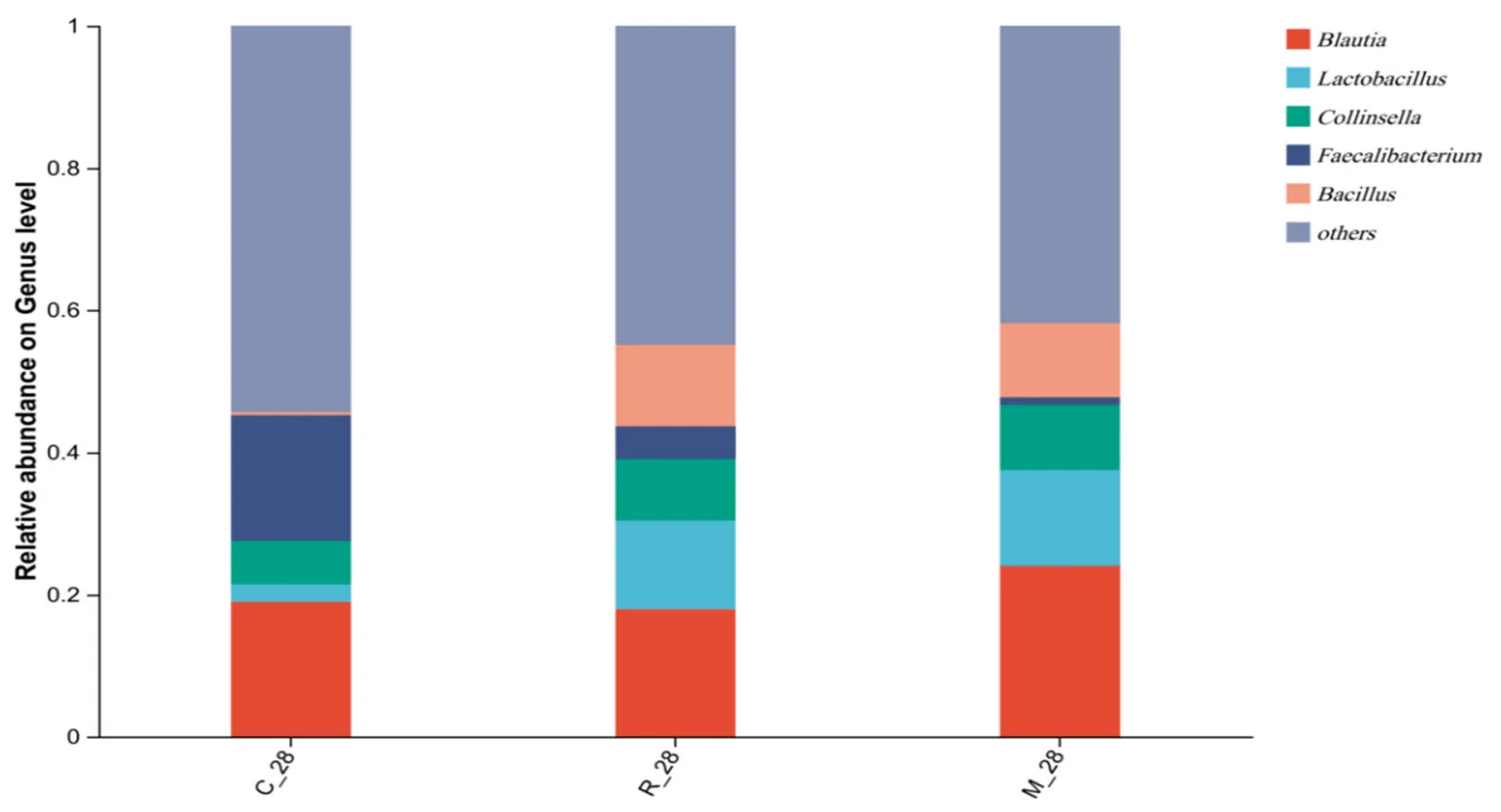
Relative abundance of microbiota at the genus level among groups. C: control; M: colon-FMT; R: colostrum-FMT.

**Figure 3:**
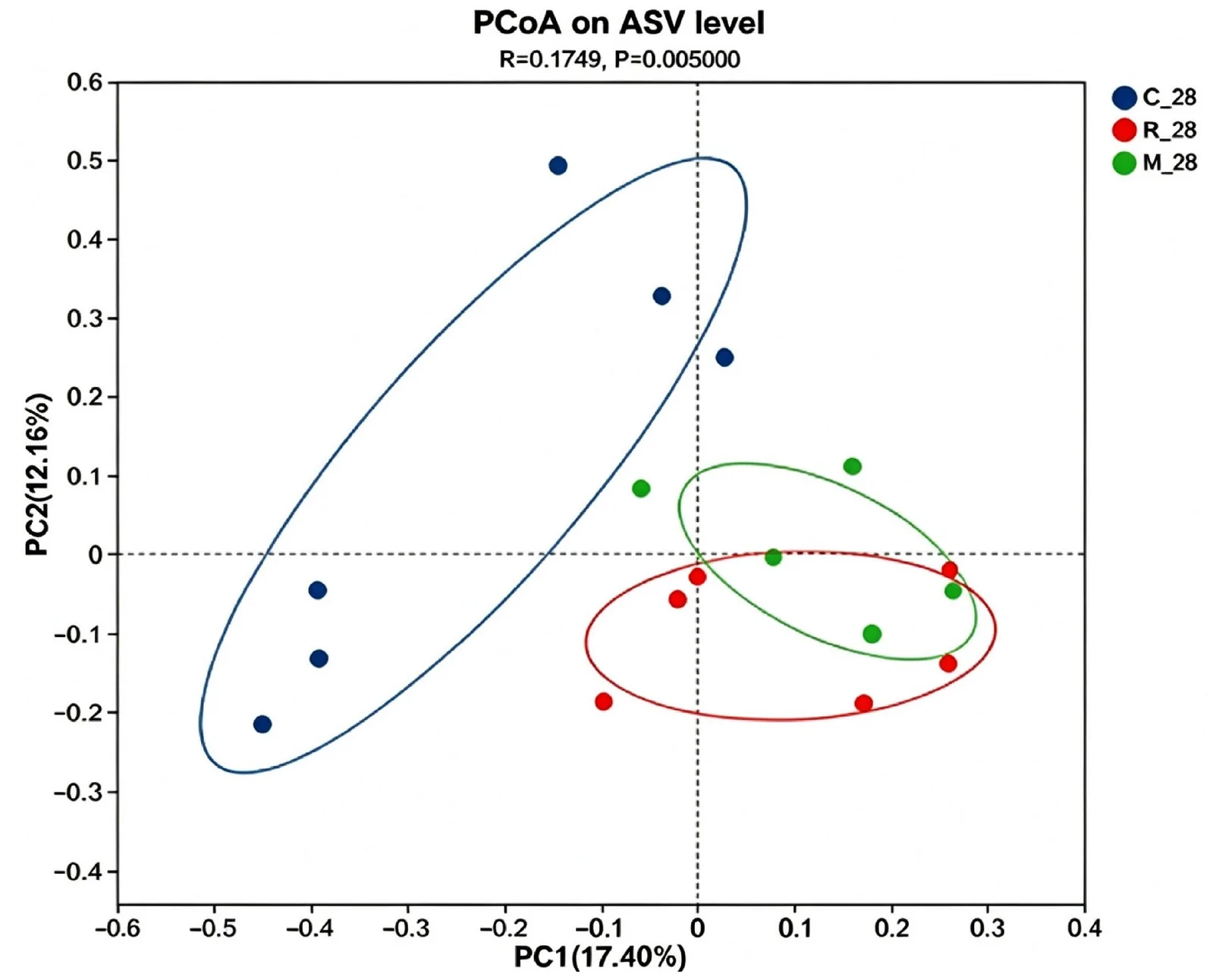
β-diversity PCoA at the ASV level among groups. C: control; M: colon-FMT; R: colostrum-FMT.

**Figure 4:**
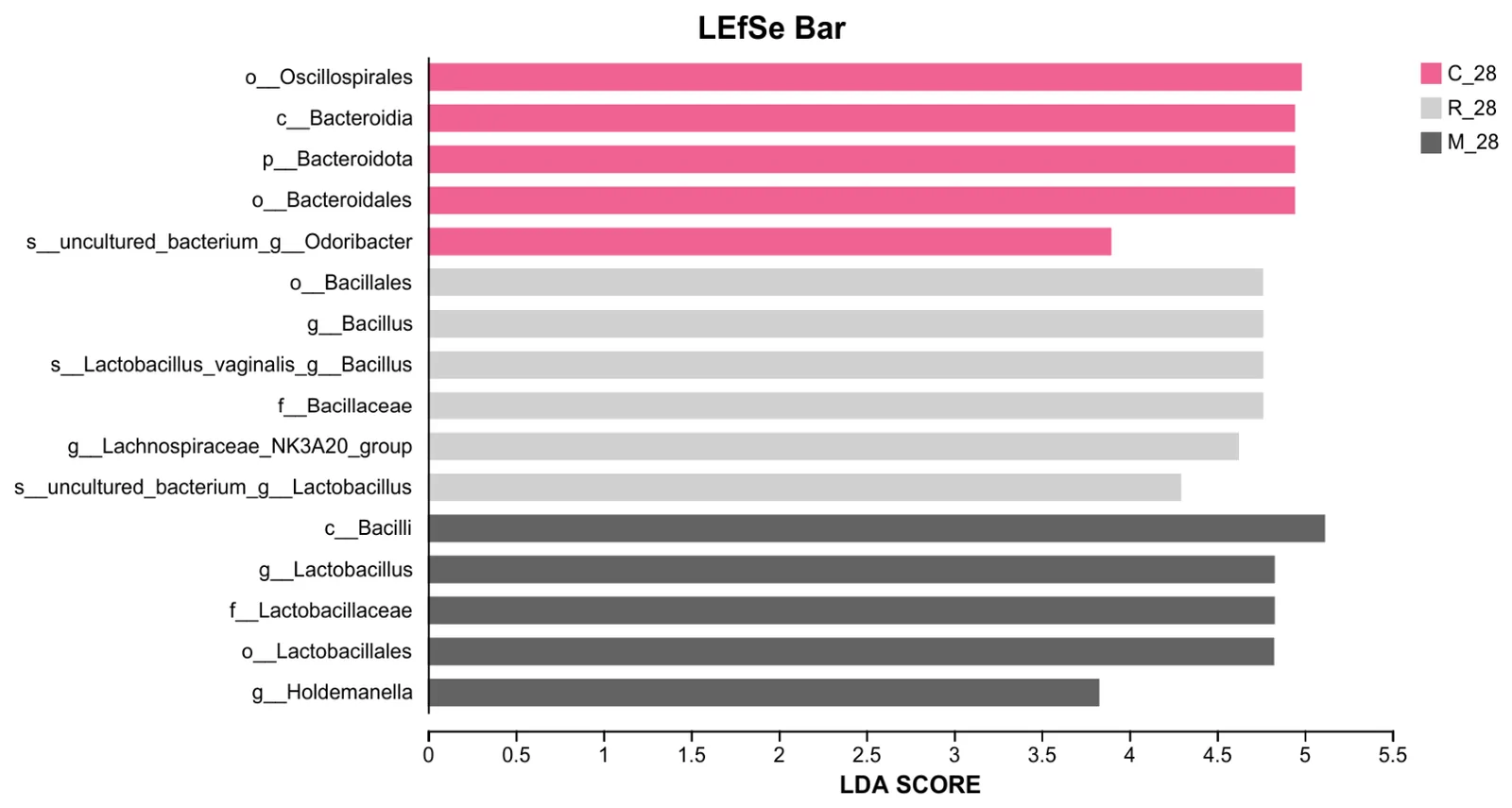
LDA score distribution of differential microbiota among groups. C: control; M: colon-FMT; R: colostrum-FMT.

**Figure 5:**
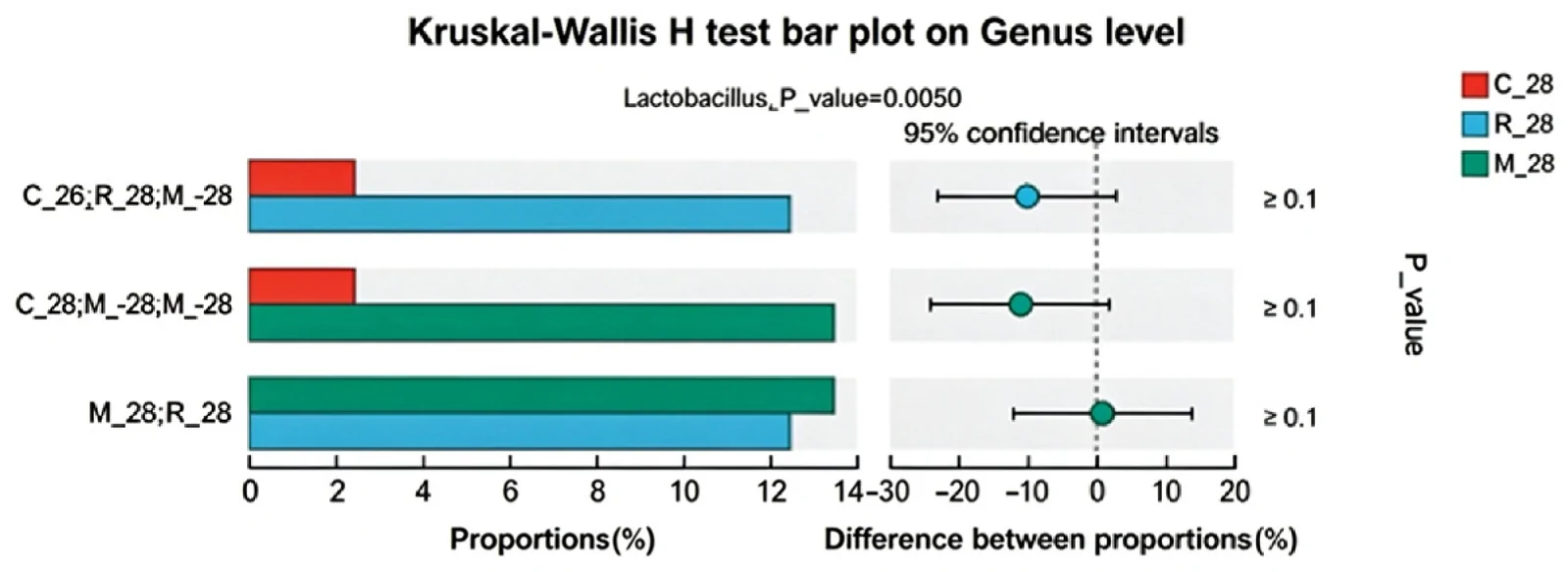
Relative abundance of *Lactobacillus* at the genus level among groups. C: control; M: colon-FMT; R: colostrum-FMT.

Donor fecal microbiota was dominated by phylum *Firmicutes* and genus *Blautia*, which collectively accounted for over 80% of total sequences. At the phylum level, *Firmicutes*, *Actinobacteriota* and *Bacteroidota* were predominant across all calf samples (Figure 1). *Bacteroidota* abundance was significantly lower in the colon group (M-28) compared with the control (C-28) and colostrum group (R-28), whereas the relative abundances of *Firmicutes*, *Proteobacteria* and *Cyanobacteria* did not differ among treatments (*p* > 0.05). The dominant genera were *Blautia* and *Lactobacillus* (Figure 2). PCoA combined with PERMANOVA based on ASV data showed no obvious divergence in overall gut microbial community structure across groups (*p* > 0.05), demonstrating that FMT altered only specific taxa rather than the whole microbial profile (Figure 3). LEfSe-LDA analysis identified four genera (*Bacillus*, g_*Lachnospiraceae NK3A20 group*, *Lactobacillus*, *Holdemanella*) as key biomarkers differentiating the three experimental groups (Figure 4). Further pairwise genus-level comparisons revealed that the colon group had markedly higher *Lactobacillus* abundance than the control and colostrum groups at D28 (*p* < 0.05, Figure 5). In addition, *Bacillus* relative abundance was significantly lower in control group compared with both FMT-treated groups (*p* < 0.01); *Faecalibacterium* tended to be higher in the colon-FMT group (*p* = 0.06). Microbiota sequencing was performed only for D28 fecal samples; we lack microbiota profiles for the D29-56 window in which key phenotypic divergence occurred.

**Figure 6:**
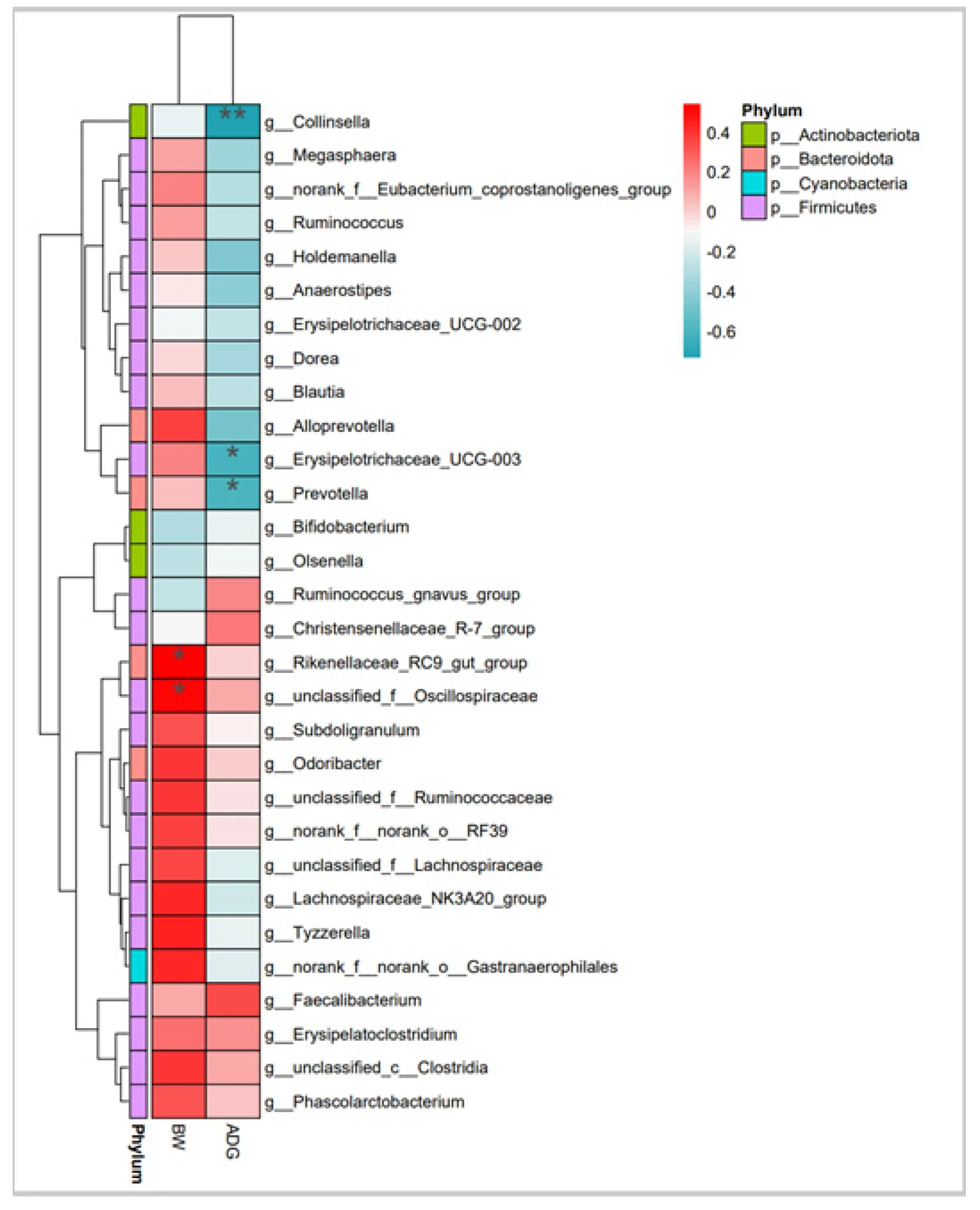
Correlation analysis of gut microbiota genera with BW and ADG. * 0.01 < *p* ≤ 0.05, ** *p* ≤ 0.01.

At the genus level, three taxa were significantly negatively correlated with ADG (*p* < 0.05): *unclassified_f_Lachnospiraceae*, *Alloprevotella*, and *Ruminococcus*. Two genera, *Erysipelotrichaceae_UCG-002* and *Blautia*, exhibited positive correlation trends with BW, although these associations did not reach statistical significance (*p* > 0.05), consistent with the absence of significance markers in Figure 6. These correlation analyses are exploratory, and no formal multiple-testing correction was applied.

## 4. Discussion

### 4.1. Effects of FMT on Growth Performance of Pre-Weaned Calves

Early calf growth determines lifelong productivity and breeding economic benefits; BW reflects general health, while ADG directly characterizes growth rate. Early gut microbiota intervention via FMT may improve growth performance by reconstructing intestinal microecology and enhancing microbial metabolic capacity, consistent with previous piglet [35,36,37,38] and ruminant [31,32] studies.

Maternal FMT elevated ADG in newborn piglets [36,37]; similarly, FMT increased body weight in beef calves and alleviated diarrhea in low-birth-weight milk-fed calves, with persistent weight advantages lasting to 12 months of age [31,32].

In our trial, final BW showed no intergroup differences, but colon-FMT calves exhibited significantly higher ADG during D29-56. We hypothesize FMT-derived beneficial microbes and growth-promoting metabolites exert major effects in the late suckling stage, while the limited trial period failed to induce significant disparities in cumulative body weight. We note the experimental gap: there are no intermediate sampling points between D28 and D56; we cannot determine when this growth divergence emerged. In addition, we observed an apparent dissociation: colostrum-FMT exhibited worsened late-period intestinal health without corresponding statistically significant reduction in ADG over D29-56.

Notably, deteriorated gut health, indicated by higher diarrhea and fecal scores in the colostrum-FMT group during D29–56, was not accompanied by reduced ADG over the same period. One plausible explanation is that the intestinal perturbations were predominantly moderate in magnitude and did not cause marked depression of feed intake. Calves may establish physiological compensation to cope with gut disturbance, which could mask potential negative impacts on growth performance. Still, this apparent disconnect between gut-health-related phenotypes and ADG should be acknowledged when interpreting the present results.

### 4.2. FMT Remodels Calf Intestinal Microbial Profiles

No differences in alpha diversity were detected across groups at the ASV level (*p* > 0.05), meaning FMT altered microbial composition without changing overall community richness or evenness. *Firmicutes*, *Actinobacteriota* and *Bacteroidota* dominated all calf gut samples, consistent with typical ruminant core microbiota [30]. Colon-FMT significantly elevated *Bacteroidota* abundance without changing *Firmicutes* levels; balanced ratios of these two core energy–metabolic phyla improve fermentation substrate utilization efficiency [39].

*Blautia*, *Bifidobacterium*, *Bacteroides* and *Lactobacillus* were the dominant genera. As a ubiquitous anaerobic taxon, *Blautia* produces acetate to nourish intestinal epithelium and suppress inflammation, and its high abundance in donor feces lays a favorable microbial foundation for FMT. LEfSe-LDA identified *Bacillus*, g_*Lachnospiraceae* NK3A20 group, *Lactobacillus* and *Holdemanella* as signature biomarkers, proving FMT selectively enriches functional beneficial genera. Several bacterial taxa were identified as potential biomarkers by LEfSe analysis, including *Bacillus*, *Lachnospiraceae NK3A20* group, *Lactobacillus*, and *Holdemanella*. Members of *Bacillus* and *Lactobacillus* are widely regarded as candidate probiotic taxa capable of modulating intestinal barrier function and immune responses in ruminants. *Lachnospiraceae NK3A20* group participates in short-chain fatty acid biosynthesis and contributes to gut carbohydrate fermentation. *Holdemanella* is involved in fatty acid metabolism and has been linked to host inflammatory status. Variations in the relative abundance of these genera may therefore partially reflect shifts in gut immune and metabolic homeostasis following colostrum-FMT intervention.

Correlation analysis linked specific taxa to growth traits: *Blautia* and *Erysipelotrichaceae_UCG-002* were positively correlated with BW, while *unclassified_f_Lachnospiraceae*, *Alloprevotella* and *Ruminococcus* were negatively correlated with ADG. These associations are exploratory and not causal proof. Of note, we cannot confirm whether D28 microbial profiles persisted through D29-56 to drive later-phase phenotypes, as no D56 microbiota data are available. Colon-FMT enriched growth-favorable *Blautia* at D28 and suppressed growth-inhibitory taxa, which could contribute to boosting late-stage energy utilization and weight gain, whereas colostrum-FMT failed to remodel these functional genera at D28, leading to lower D29-56 ADG.

### 4.3. Enrichment of Beneficial Genera Improves Intestinal Health

The colon-FMT group had higher *Lactobacillus* abundance than the other two groups (*p* < 0.05), matching its reduced late-stage fecal scores (*p* < 0.01). *Lactobacillus* secretes lactic acid to lower gut pH, inhibit pathogens, strengthen mucosal barriers and relieve inflammation, and its abundance has been negatively correlated with calf diarrhea incidence, as reported in piglet research [40].

Colon-FMT calves also harbored more *Faecalibacterium*, a key butyrate producer that maintains intestinal homeostasis. The control group had lower *Bacillus* abundance than both FMT groups (*p* < 0.01); *Bacillus* balances gut microbiota and enhances host immunity. Both delivery routes raised intestinal *Bacillus*, yet the synergistic regulatory interaction between FMT and *Lactobacillus* requires further exploration.

Donor feces were dominated by *Firmicutes* and *Blautia*. Some donor-associated taxa were detectable in FMT-treated calves at D28, indicating possible transfer of donor-derived bacteria. However, in the absence of quantitative similarity metrics and serial sampling, the extent and persistence of donor microbe engraftment remain to be determined. Further dedicated studies are required to quantitatively evaluate donor microbe engraftment efficiency in neonatal calves following FMT.

### 4.4. FMT Modulates Systemic Immunity via Gut Microbiota Remodeling

Gut dysbiosis is the primary cause of neonatal calf diarrhea, and FMT restores balanced microbiota via healthy donor microbial communities. The superior intestinal health of colon-FMT calves was closely associated with enriched beneficial genera such as *Lactobacillus*, which suppress pathogens and regulate immune mediator secretion to maintain host homeostasis.

Colon-FMT calves tended to have higher serum IgG concentrations at D14 compared with the control and colostrum-FMT groups (*p* = 0.05). This borderline result does not constitute robust evidence for a treatment-driven effect; thus, this finding should be interpreted cautiously and not overemphasized. At D56, their serum IL-2 and IL-4 concentrations were also greater than those of the colostrum-FMT group (*p* ≤ 0.05). IL-2 and IL-4 are marker cytokines of mutually inhibitory Th1/Th2 cells that maintain gut immune balance: IL-2 eliminates intracellular pathogens, and IL-4 mediates mucosal humoral immunity [41]. Th1/Th2 imbalance causes intestinal inflammation and calf diarrhea [42]. Unchanged IL-6 and IL-10 across groups indicate FMT modulates immunity predominantly through the Th1/Th2 axis, rather than through acute inflammation or IL-10-related anti-inflammation. Overall, colon FMT reshapes gut flora to coordinate systemic and mucosal immunity, forming a microbe–immunity–health regulatory network in pre-weaned calves.

### 4.5. Divergent Effects of Two FMT Delivery Routes

The two FMT modes induced distinct gut microbial shifts. Colostrum-FMT reduced diarrhea incidence during D15-28 (*p* < 0.05) but caused elevated diarrhea rates in late stages (D29-56, *p* < 0.01), accompanied by failed enrichment of intestinal Lactobacillus at D28. We propose a testable hypothesis that colostrum bioactive substances (immunoglobulins, oligosaccharides) offer short-term immune protection but hinder transplanted microbial colonization, disrupting sustained microecological improvement and impairing long-term intestinal health. Reduced IL-2 and IL-4 at D56 reflect disrupted mucosal T-cell homeostasis and attenuated Th2 responses, which may compromise gut barrier function and partly explain late diarrhea in colostrum-FMT calves without elevated pro-inflammatory cytokines. These speculative findings await further mechanistic validation.

The FMT delivery route determines the donor microbial colonization efficiency and persistent efficacy. Unlike colostrum perfusion, colonic administration avoids interference from colostrum matrices, potentially supporting stable beneficial bacterial colonization and long-term gut homeostasis in pre-weaned calves.

### 4.6. Limitations

Important study limitations include the moderate sample size, single-farm Holstein cohort, absence of intermediate sampling between D28 and D56, and microbiota profiling only at D28. Key phenotypic divergence emerged in the D28-D56 window, but the sampling gap limits our ability to pinpoint the time point when the altered growth performance and reversed diarrhea phenotype of the colostrum-FMT group occurred. The D28 microbiota snapshot cannot directly explain late-phase D56 phenotypes. Our findings are associative rather than definitive mechanistic evidence. Further multi-farm, multi-time-point trials are required to validate the engraftment dynamics and real-world applicability of colonic-route FMT in neonatal calves.

## 5. Conclusions

Colostrum-period colonic FMT is associated with enrichment of intestinal beneficial *Lactobacillus*, improved Th1/Th2 immune balance, enhanced late-stage weight gain and reduced long-term diarrhea in pre-weaned calves. Although colostrum-delivered FMT relieves early diarrhea, it fails to sustain growth and intestinal benefits due to poor colonization of donor microbes. The FMT delivery route appears to determine its persistent efficacy in neonatal calves. Colonic FMT represents a promising research direction for non-antibiotic gut health intervention, and this study provides microbial evidence linking gut microbiota to growth and immunity in Chinese Holstein calves.

## Figures and Tables

**Table 1:** Effect of FMT on growth performance of calves.

Item	Control	Colon-FMT	Colostrum-FMT	SEM	*p*-Value
BW, kg					
D1	38.78	41.31	40.19	0.86	0.27
D7	43.75	43.94	45.63	0.83	0.40
D14	48.09	49.69	49.97	0.72	0.33
D28	59.19	59.13	60.56	0.84	0.52
D56	90.56	90.31	88.09	1.06	0.38
ADG, kg/d					
D1–D7	0.84	0.44	0.76	0.06	0.07
D8–D14	0.79	0.79	0.67	0.04	0.49
D15–D28	1.12	1.11	0.98	0.03	0.19
D29–D56	1.19 ^b^	1.23 ^a^	0.98 ^b^	0.04	0.01
D1–D56	0.81	0.87	0.78	0.03	0.49

ADG: group ADG represents the arithmetic mean of individual ADG within each treatment. SEM: standard error of the mean. ^a,b^: means with different superscripts within a row differ significantly (*p* < 0.05).

**Table 2:** Effects of FMT on calf intestinal health.

Item	Control	Colon-FMT	Colostrum-FMT	SEM	*p*-Value
Fecal scores					
D1–D7	1.45	1.16	1.24	0.05	0.06
D8–D14	1.86	1.82	1.77	0.06	0.84
D15–D28	1.76	1.73	1.42	0.07	0.07
D29–D56	1.42 ^b^	1.54 ^b^	1.77 ^a^	0.04	0.003
Diarrhea rate					
D1–D7	0.19	0.10	0.08	0.02	0.08
D8–D14	0.22	0.22	0.15	0.02	0.29
D15–D28	0.22 ^a^	0.20 ^a^	0.08 ^b^	0.02	0.01
D29–D56	0.12 ^b^	0.11 ^b^	0.17 ^a^	0.01	0.007

SEM: standard error of the mean. ^a,b^: means with different superscripts within a row differ significantly (*p* ≤ 0.05).

**Table 3:** Effects of FMT on serum immunoglobulin levels.

Item	Control	Colon-FMT	Colostrum-FMT	SEM	*p*-Value
IgG, μg/mL					
D7	39.58	37.00	43.25	2.39	0.59
D14	51.17 ^b^	62.92 ^a^	57.12 ^b^	2.03	0.05
D28	53.76	55.61	57.93	1.74	0.64
D56	54.78	55.03	57.83	1.80	0.76
IgA, μg/mL					
D7	14.46	13.33	16.86	0.83	0.22
D14	17.02	18.52	17.77	0.67	0.68
D28	18.20	20.10	20.30	0.71	0.43
D56	19.01	19.42	21.15	0.82	0.54
IgM, μg/mL					
D7	5.48	6.45	6.96	0.33	0.19
D14	7.25	7.75	7.74	0.32	0.77
D28	11.55	10.66	11.99	0.34	0.27
D56	12.05	10.59	12.17	0.43	0.26

SEM: standard error of the mean. ^a,b^: means with different superscripts within a row differ significantly (*p* < 0.05).

**Table 4:** Effects of FMT on serum Interleukin levels.

Item	Control	Colon-FMT	Colostrum-FMT	SEM	*p*-Value
IL-2, pg/mL					
D1	1164.61	1287.06	1107.29	37.12	0.13
D7	1140.16	1202.18	1100.16	22.87	0.19
D14	1083.31	1025.77	982.13	20.20	0.12
D28	964.38	960.09	868.65	18.73	0.06
D56	818.73 ^a^	763.99 ^a^	675.52 ^b^	19.40	0.004
IL-4, pg/mL					
D1	592.24	624.49	591.08	10.56	0.35
D7	572.86	605.84	542.17	12.45	0.12
D14	558.54	528.68	533.89	10.55	0.48
D28	539.32	506.75	496.79	12.74	0.38
D56	519.86 ^a^	504.92 ^a^	465.05 ^b^	9.84	0.05
IL-6, pg/mL					
D1	231.24	265.47	214.41	14.06	0.33
D7	198.08	186.71	191.16	14.60	0.95
D14	209.02	176.35	184.58	14.16	0.64
D28	229.02	273.29	242.45	16.62	0.55
D56	264.38	244.65	223.32	11.92	0.38
IL-10, pg/mL					
D1	341.21	366.70	291.83	14.02	0.08
D7	280.70	341.46	261.80	20.74	0.27
D14	299.96	281.78	230.17	16.83	0.22
D28	362.42	260.46	305.95	13.25	0.11
D56	304.55	248.46	261.43	18.83	0.46

SEM: standard error of the mean. ^a,b^: means with different superscripts within a row differ significantly (*p* < 0.05).

## Data Availability

The datasets generated and analyzed during the present study are available from the joint corresponding authors upon reasonable request. The raw data are not publicly available due to relevant research group restrictions.
